# Anti-asthma medication prescribing to children in the Lombardy Region of Italy: chronic versus new users

**DOI:** 10.1186/1471-2466-11-48

**Published:** 2011-10-17

**Authors:** Marina Bianchi, Antonio Clavenna, Marco Sequi,, Angela Bortolotti, Ida Fortino, Luca Merlino, Maurizio Bonati

**Affiliations:** 1 Laboratory for Mother and Child Health, Department of Public Health, Mario Negri Pharmacological Research Institute, Milan, Italy; 2 Regional Health Ministry, Lombardy Region, Milan, Italy

**Keywords:** asthma, anti-asthmatic drugs, children and adolescents, pharmacoepidemiology, prescription, drug utilization

## Abstract

**Background:**

Although anti-asthma medications are amongst those most frequently under or over prescribed it is generally accepted that prescriptions for such agents can be used as a proxy for disease prevalence. The aims of this study were to estimate prevalence and incidence of childhood asthma in a representative Italian area by analysing three years of anti-asthmatic prescriptions and hospitalizations of subjects with chronic or first time treatment, and to underline appropriateness of therapeutic choices.

**Methods:**

The analysis involved prescriptions given to 6-17 year olds between 2003 and 2005 in Italy's Lombardy Region. The youths were classified as potential asthmatics, based on the different degree of drug utilization: occasional, low or high users, and grouped as 'new onset' or 'chronic' cases based on the duration of therapy dispensed. The analysis of prescriptions and hospitalization rate of these groups provided an estimate of the 2005 asthma prevalence and incidence and allowed an estimation of the level of appropriateness of treatments.

**Results:**

During 2005, the estimated incidence of potential asthmatics was 0.8% and the estimated prevalence was 3.5%. When viewed retrospectively for two years, records showed that 47% of potential asthmatics received prescriptions also during 2004 and 30% also during 2003. During the three years considered, 7.5%, 2.8%, and 1.5% of high, low, and occasional users, respectively, were hospitalized for asthma. The most important inappropriateness found was the prescription of long acting beta adrenergics as first time treatment.

**Conclusions:**

This study allowed a proxy of asthma incidence, prevalence, and severity. The analyses highlighted a low compliance with the guidelines, suggesting that educational interventions are needed to obtain a more rational management of childhood asthma, especially in subjects starting therapy.

## Background

Asthma is one of the leading chronic childhood diseases, and its prevalence in Italy remained largely stable between 1995 and 2002 (9%) [1]. Prior to school age many children experience recurrent episodes of wheezing and cough. These symptoms are frequently transient, and 60% of preschoolers are asymptomatic by age six [2,3]. For this reason, children have a higher overall prevalence of anti-asthmatic prescriptions compared to adolescents. Regarding asthma therapy, international guidelines recommend inhaled corticosteroids (ICS) for long-term control of persistent asthma and short acting β2 adrenergic (SABA), such as salbutamol, as first choice in acute attacks [4-6]. In particular, the pharmacological therapy for long term control is recommended in a stepwise approach, based on asthma severity. Although adherence to guidelines reduces the number of outpatient and emergency department visits [7], guidelines seem far from being routinely applied in clinical practice. The main inadequacy seems to be the use of steroids: over-prescribed in upper respiratory tract infections [8] and under-prescribed for preventive therapy between asthma attacks. Another inadequacy is the use of long acting beta adrenergics (LABAs). Guidelines suggest increasing the dose of ICS before adding LABAs. The FDA recommends the use of LABAs without an ICS for the shortest amount of time required to achieve control of symptoms, because of the increased risk of asthma exacerbations, hospitalizations, and death [9]. The aims of this study were to estimate prevalence and incidence of childhood asthma in a representative Italian area by analysing three years of anti-asthmatic prescriptions and hospitalizations of subjects with chronic or first time treatment, and to underline appropriateness of therapeutic choices.

## Methods

The analysis involved all paediatric prescriptions reimbursed by the National Health Service (NHS) and dispensed by the retail pharmacies of 15 Local Health Units (LHUs) in the Lombardy Region between January 2003 and December 2005. The database stores all community (i.e. outside hospital) prescriptions reimbursed by the NHS and issued to individuals living in the Lombardy Region. Prescriptions issued to the entire paediatric population are fully reimbursed by the NHS in Italy. The structure of the database has been described in detail elsewhere [10]. Although it is an administrative database, it has shown high accuracy in other studies [11].

Data were managed and analysed using an anonymous patient code. The approval by the ethics committee is not required in Italy for this kind of study.

The study population was composed of 1,016,683 children and adolescents 6-17 years old, male/female ratio 1.1, living in the Lombardy Region. The study sample represented 15% of the overall Italian paediatric population.

In 2005 in the Lombardy region there were 1165 primary care paediatricians and 6791 general practitioners. Anti-asthmatics were classified as drugs belonging to the R03 main therapeutic group of the Anatomical Therapeutic Chemical classification system (ATC).

Prevalence data by age and sex were calculated as number of children to whom anti-asthmatic drugs were dispensed per 100 inhabitants.

### Definition of potential asthmatics (PA)

Potential asthmatics (PA) were considered to be subjects ≥ 6 years old receiving, during 2005, at least one package (canister or box) of the active substances listed in Table 1.

**Table 1:** Anti-asthmatic agents

Active substances	Formulation
**SABA **(Salbutamol, fenoterol);	
**ICS **(beclomethasone, fluticasone, flunisolide, budesonide);	
**LABA **(formeterol, salmeterol, fixed association budesonide/formoterol, fixed association fluticasone/salmeterol);	Metered dose inhalers Dry powder inhalers
**Chromones **(Cromolyn Sodium, Nedocromil Sodium);	
**Anticholinergics **(ipratropium bromide, oxitropium bromide).	

**Theophyllines**;	
**LTRA **(montelukast, zaphirlukast), not as exclusive therapy;	Oral
**Steroids**, not as exclusive therapy.	

Subjects receiving exclusively anti-asthmatic drugs in nebulised formulations (appropriate under age 2) or LTRA as exclusive therapy (prescribed for allergic rhinitis control), without any other anti-asthmatic drug, were not included.

The strategy (based on anti-asthmatic prescriptions) for PA identification used in this study has been previously validated [12] by the comparison with paediatricians' diagnosis of the same subjects identified as PA. Sensitivity was 0.91 [95% confidence interval (95% CI): 0.67-1.00], whereas specificity was 0.98 (95% CI: 0.96-1.00).

### Degree of consumption

PA patients were divided into three groups, based on number of packages (canister or box) received during 2005: A) "occasional users", subjects receiving only one package (i.e. a single course of therapy); B) "low users", subjects receiving 2-4 packages; C) "high users", subjects receiving 5 or more packages. The threshold of five packages was chosen because it represents the 90th percentile in distribution of frequency of treated children by number of packages.

### 'Chronic' and 'new onset' cases

In order to evaluate the extent of chronic treatment, people who received anti-asthmatic drug prescriptions also during 2003 and 2004 were identified. Moreover, patients with new onset asthma were described as: 1) not having received anti-asthmatic therapy during the previous two years (2003 and 2004) and 2) not having been hospitalized for asthma during the 2003-2005 period. A comparison of prescription profiles between chronically treated youths (three year persistence of therapy) and youths with new onset asthma was performed. A chi-square test (χ ^2^) was performed between groups in order to evaluate statistically significant differences.

### Hospitalizations

Rate of hospitalization for asthma (corresponding to the diagnosis code 493 of the International Classification of Diseases ICD-9-CM ) in children 6-17 years old was estimated for the period from January 2003 to December 2005 using hospital discharge forms.

### Differences between the LHUs

Estimation of the prevalence of asthma at LHU level was calculated as the number of PA per 100 children aged 6-17 who were listed under that particular LHU. Estimation of the incidence of asthma at LHU level was calculated as the number of PA youths who received an anti-asthmatic prescription for the first time in 2005 per 100 children aged 6-17 who were listed under that particular LHU. The relationship between incidence and prevalence and between prevalence and hospitalization rates by local health unit was investigated using the nonparametric Spearman rank correlation test. Finally, a logistic regression analysis was performed in order to identify risk factors for asthma by evaluating the association between drug prescription and gender, LHU of residence, kind of physician in charge of the patient (paediatrician vs. general practitioner), and physician gender. Concerning the LHU of residence, Milan was chosen as reference, since a previous study found it had the LHU with the lowest prevalence of drug prescriptions [11]. The odds ratio (OR) and relative 95% confidence interval (CI) between the groups were calculated. Statistical analysis was performed using SAS software, version 9.1 (SAS Inc., Cary, NC, USA). A *p *value < 0.05 was considered to be statistically significant.

## Results

### Prevalence and incidence of potential asthmatics (PA)

Potential asthmatics were 35,399 (3.5% of the 6-17 year old population). The median age, interquartile range (IQR), and boy/girl ratio were, respectively 11, 9-14, and 1.7. The occasional users were 9,482 (27% of the PA), the low users were 16,438 (46%), and the high users were 9,479 (27%). During the three years considered, 2,456 children and adolescents were hospitalized at least once for asthma. Among these subjects, 1318 (54%) were identified as PA, whereas 1138 (46%) did not receive any anti-asthmatic drugs during 2005 or received nebulised formulation or montelukast as exclusive treatment. The percentage of PA children hospitalized for asthma ranged from 1.5% in the occasional users to 2.8% in low users, and 7.5%, in the high users ($\chi_{t}^{2}=487$; *p *< 0.001), while the hospitalization rate for other reasons was constant in the three groups. In all, 16,629 children and adolescents (47% of the subjects defined PA) received age-appropriate anti-asthmatic prescriptions also during 2004 and 10,712 (30%) during 2004 and 2003 (Table 2). The median age of these chronically treated patients was 11 (IQR = 8-14), with a higher prevalence in boys. Most children were cared for by a family paediatrician (Paediatrician/GP ratio = 1.2). The percentage of chronically treated patients differed between the three groups of users, and ranged from 45% in occasional users to 87% in high users. ($\chi_{t}^{2}=3636$; *p *< 0.001).

**Table 2:** Percentage of children and adolescents identified as new onset or chronic asthmatics and their anti-asthma therapy

DRUG CLASSES	NEW ONSET	CHRONIC ASTHMA
	8,058	10,712
***Rescue Medications***		
**SABA**	5,023 (62)	7,121 (56)
SABA exclusively^a^	1,933 (24)	1,138 (11)
**Systemic steroids**	391 (5)	919 (9)

***Controller Medications***		
**ICS**	4,876 (44)	8,569 (56)
alone^b^	2,661 (33)	3,176 (30)
ICS+LABA	1,884(7)	4,579 (19)
ICS+LTRA	267 (3)	1,181 (11)
ICS+Others^c^	902 (11)	2,848 (27)
**no ICS**	610 (8)	643 (6)
LABA	130 (2)	82 (0.3)

^a ^without other anti-asthmatic prescriptions

^b ^without other controller drug prescriptions

^c ^Others = not ICS, not SABA, not LABA, not LTRA

data are expressed as number of subjects (% of each population)

A total of 8,058 subjects (23% of the subjects defined as PA) were diagnosed as potential asthmatics for the first time during 2005 (incidence 0.8%). The median age was 12 (IQR = 9-15), and the boy/girl ratio was 1.4. The majority of children were cared for by a general practitioner (Paediatrician/GP ratio = 0.7).

### Prescription profiles

In all, 66% of the PA subjects were treated with SABA (1/5 of these received SABAs exclusively), 36% with ICS alone, 45% with ICS and other anti asthmatics (23% with ICS and LAB A), and 6% with other anti asthmatics. 95% of PA subjects received inhaled formulations and 16% of children and adolescents also received oral formulations (theophyllines, LTRA, or steroids). The drug most commonly prescribed was salbutamol (55% of subjects), followed by fluticasone (29%), beclomethasone, and salmeterol/fluticasone in fixed combination (both 23%). 6.7% received systemic steroids, with differences between groups, with the high users having nearly three times a greater chance of receiving systemic steroids compared to occasional users (11.5% vs. 4.0%, respectively; χ ^2 ^= 430; *p *< 0.001). The comparison between prescription profiles of chronically treated youths and youths with new onset asthma is reported in Table 2. The percentage of youths receiving at least one SABA prescription was similar in chronically treated users and in new users (56% versus 62%), while the percentage of youths receiving SABA as exclusive therapy was higher in new users than in chronically treated users (24% versus 11%). The percentage of children and adolescents receiving ICS alone was similar (30% of chronic versus 33% of new users). On the contrary, new users receiving ICS with other anti-asthmatics (LABA, LTRA, or others) were fewer than chronically treated patients. Furthermore, the percentage of children with at least one prescription of systemic steroids was higher in those chronically treated than in new users (9 versus 5%).

### 'New onset' cases and first drugs received

In all, 45% of the youths with new onset asthma received only one medication package (occasional users), while only 9% resulted as high users (Table 3). In all, 7% of new users received LABA associated with ICS and 2% without ICS (Table 2). Salbutamol was the drug most commonly given as the first anti-asthmatic prescription (45% of youths with new onset asthma), followed by beclomethasone (17%), fluticasone (14%), and salmeterol/fluticasone (13%). In all, 20% of the patients with new onset asthma received 2 or more drugs as their first prescription (mainly salbutamol and beclomethasone). A total of 614 youths (8% of the new onsets) received only salmeterol and fluticasone in fixed combination as their first and unique prescription (occasional users). Moreover, 66 children received only one prescription of LABA without any other anti-asthmatic.

**Table 3:** Prescriptions to new onset asthmatic children and adolescents

DRUG CLASSES	Occasional	Low	High	Total
	3,621	3,752	685	8,058
***Rescue Medications***				
**SABA**	1,986 (55)	2,479 (66)	558 (81)	**5,023 (62)**
SABA exclusively	1,529 (42)	395 (10)	9 (1.3)	**1,933 (24)**
not receiving SABA	1,635 (45)	1,273 (34)	127 (19)	**3,035 (38)**
**Systemic steroids**	127 (3.5)	196 (5)	68 (10)	**391 (5)**

***Controller Medications***				
**ICS**	1,368 (38)	2,874 (77)	634 (92)	**4,876 (61)**
ICS alone	754 (21)	1,703 (45)	204 (30)	**2,661 (33)**
ICS+LABA	614 (17)	941 (25)	329 (48)	**1,884 (23)**
ICS+LTRA	0	118 (3)	149 (22)	**267 (1)**
ICS+Others	0	584 (16)	318 (46)	**902 (11)**
**No ICS**	267 (7.4)	304 (8)	39 (6)	**610 (7.6)**
LABA alone	66 (1.8)	64 (1.7)	0	**130 (1.6)**

data are expressed as number of subjects (% of each population)

### Differences between the LHUs

**Figure 1:**
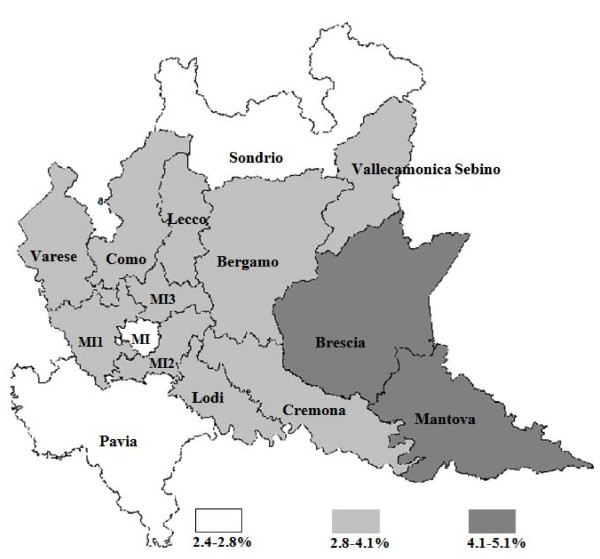
**Prevalence of R03 prescriptions in potential asthmatic children and adolescents in the 15 LHUs in the Lombardy region during 2005**.

Large differences were found in prevalence of PA rates between different LHUs, ranging from 2.5% in Pavia to 5.1% in Mantova (Figure 1). Differences in prevalence of asthma persist also after adjusting for gender and age. A similar geographical distribution was found for the rate of chronically treated youths, with a prevalence ranging from 0.7% in Pavia to 1.8% in Mantova. A statistically significant correlation between rank distributions at LHU level of the total PA and chronically treated youth prevalence rate was found (r_s _= 0.92; *p *= 0.0006). Small differences in the incidence of asthma (percentage of youths with new onset asthma) between LHUs were found; these ranged from 0.59% in Pavia to 0.94% in Como. No correlation was found between the rank distribution at LHU level of incidence and prevalence of PA, nor between incidence and percentage of chronically treated youths. Moreover, no correlation was found between rank distribution at LHU level of the PA prevalence rates and hospitalizations during the 2003-2005 period (ranging from 0.1% in Bergamo to 0.4% in Lodi).

Being male (OR 1.67, 95% CI 1.64-1.71) and living in Mantova (OR 1.91, 95% CI 1.80-2.03) were the factors associated with the highest chance of being identified as a PA subject. On the contrary, youths living in Pavia had a lower chance of being asthmatic compared with children living in Milan (OR 0.91; 95%CI 0.85-0.97).

## Discussion

It was possible to define the severity of asthma in children and adolescents 6-17 year old based on the quantity and quality of the therapy by using a database that stores all community prescriptions of anti-asthmatic drugs.

The main finding of this study is the inappropriateness of drug prescriptions, especially in the prescription of LABAs without steroids. In particular, it seems that the subjects who are new cases of asthma are the most inappropriately treated: 38% does not receive SABA, 8% does not receive ICS, 2% receives LABA alone, and 23% receives the association ICS+LABA.

### Strengths and limits

Many studies describing the actual use of asthma medication in children show a wide variability in treatment, reporting an over-use in children without asthma and an under-use in children with doctor-diagnosed asthma [13-20]. There has therefore been a debate on whether anti-asthmatic drug utilization studies are a good proxy for the disease or not [21,22]. However, the strategy applied in this study, which is similar to those in other studies [13,19,23,24], has been validated in identifying potentially asthmatic patients [12]. The limits of this study are the absence of details on prescriptions (i.e. diagnosis and dose) and the fact that the Lombardy Region is the top Italian region according to the socio-demographic and economic national profile and may thus not be fully representative of other Italian settings in which anti-asthmatic prescriptions and consumption may be different. However, the large size of the studied population made the data suitable for studying the rational use of drugs in paediatric asthma and other diseases [11]. Moreover, prescriptions issued to the entire paediatric population are fully reimbursed by the NHS in Italy and this allows the study of the whole population.

### Estimation of asthma prevalence and incidence in Lombardy

The first criteria, chosen with the aim to identify potentially asthmatic subjects, was age ≥ 6 years because only in school-aged children is it possible to diagnose asthma in a reliable manner [2,3]. The other criteria involved the prescription of age-appropriate formulations, and the use of nebulizers was therefore excluded. LTRA as monotherapy was excluded because montelukast alone is prescribed almost exclusively for allergic rhinitis control. Using these criteria, a 3.5% asthma prevalence was estimated. This prevalence is similar to that in a previous report on the population of Lecco's LHU, in which the prevalence of PA in 2003 was 3.8% [25] and is consistent with the prevalence estimated in young Italian adults [26]. It is possible that the prevalence of asthma was overestimated in the SIDRIA survey [1] since the reliability of parent-reported asthma or asthma-like symptoms, as a measure of asthma prevalence, is questionable. The classification of the population based on the number of anti-asthmatic packages received (high, low and occasional users) during 2005 seems reliable in estimating severity, since the percentage of hospitalizations for asthma increased from occasional to high user groups. Among the 2456 children and adolescents who underwent hospitalization for asthma once, only 54% were treated with age-appropriate formulations of anti-asthmatics, while 46% did not receive any anti-asthmatic treatment or received nebulised formulation or montelukast as exclusive treatment. The fact that nearly half of children and adolescents hospitalized for asthma at least once during the three year period did not receive adequate therapy for asthma, a finding consistent with other studies [15,16], may also be due to an error in the diagnosis occurring at the time of hospitalization. However, even taking into account the 1138 youths that were hospitalized, but who did not receive an appropriate anti-asthma drug therapy during 2005, and the 20,158 youths who received anti-asthma treatment during 2004, but not during 2005, the overall percentage of children having asthma episodes during 2005 or who had had episodes in the previous 12 months can be estimated at 5.6%, which is very similar to the data collected by the SIDRIA survey concerning "wheezing during the past 12 months". This finding seems to support the hypothesis that an overestimation of the asthma prevalence in the SIDRIA survey does exist when "lifetime asthma" is reported by the parents [1].

The incidence of asthma in the Lombardy region during 2005, estimated in 6-17 year old subjects, was 0.8% (Table 2), lower than the incidence in Norway (1.5% in the population of 2-29 year olds) [23] and in the Netherlands (4.3% in the 0-9 year old population) [20]. The differences are probably due to the different age groups; preschoolers in particular may increase the incidence in Norway and in the Netherlands [23,20]. As for the incidence, in the Lombardy region the prevalence was also lower (3.5%) than in Norway (5.1%) [23] and the Netherlands (8.1%) [20], probably for the same reason.

### The 'chronic' and 'new onset' cases and inappropriateness of treatments

A different prescribing pattern emerged when comparing youths receiving chronic asthma treatment with youths with new onset asthma receiving treatment for the first time (Table 2). The fact that 24% of the new users received SABA exclusively, versus 11% of chronic users, was expected. The fact that 44% of new users received ICS as unique controller therapy, versus 56% of the chronically treated patients, was also expected. Moreover, chronically treated subjects more commonly received ICS+ LABA than new users, as guidelines suggest. However, the finding that high numbers of new users received LABA alone or LABA+ICS (2 and 7%, respectively), a second line therapy that should be used in children with moderate-severe asthma who do not benefit from ICS alone [4-6], is actually raising some concerns.

This inappropriate use of LABA as initial therapy was also reported for a US paediatric population [27]. In steroid-naïve subjects, the combination of ICS and LABA did not significantly reduce the risk of exacerbations requiring rescue oral corticosteroids compared to the use of ICS alone with a similar dose [28]. Even in children already under ICS therapy, it has been reported that the addition of LABA is not associated with a significant reduction in the rate of exacerbations [29]. In a recent article [30], percentages of LABA (mis)utilization similar to this study were reported during 2005 in England. Although the use of LABA alone is not rational, the article reported an improvement of adherence to guidelines from 2000 to 2005, with a decrease of use of LABA alone and an increase of the LABA+ICS association [30]. In Table 3 the new onset users are grouped based on number of packages received, and more inadequacies are made evident: 38% of new users did not receive a SABA during the one year observation period and, looking at the first anti-asthmatic drug prescriptions given to the new cases of asthma, more than half of the subjects did not receive salbutamol. Nearly 20% of high users did not receive any SABA prescription. Most of them received ICS+other controller drugs. In all, 680 children and adolescents, 8% of subjects suffering from new onset asthma, were treated the first, and only, time (occasional users) with LABA alone (1% of new users) or with LABA associated with ICS (8% of new users).

### Prescribing differences in the 15 LHUs

Some differences between LHUs in the Lombardy region were found in the prevalence of anti-asthmatic prescriptions and the rate of PA seems to be higher in the south-eastern part of the region (Figure 1). These differences might be due to different factors: differences in prescribing attitudes (Brescia also has a high prevalence of prescription of drugs other than anti-asthmatics [11], and differences in socio-economic status or disease triggers, i.e. air pollution (Brescia and Mantova are the areas with the highest prevalence of anti-asthmatic prescriptions also in the adult population). Epidemiological studies estimated a prevalence of asthma of 10% both in Milan [31] and in Brescia [15]. On the contrary, the prevalence of asthma medications differed between the two LHUs (2.7% in Milan and 4.2% in Brescia). However, it should be noted that in Brescia the prevalence of asthma diagnosed by physicians was 10.2%, but only 41% of children were under therapy when the survey was performed [15]. Hence, the prevalence of anti-asthmatic prescriptions in Brescia found in this study (4.2%) is consistent with the data already published [15], suggesting that the model used in this study may be valid in measuring asthma prevalence, taking into consideration the under-treatment of asthma. The greatest gap between prevalence of drug prescriptions and asthma prevalence was observed in Milan, and it is possible that in this LHU a higher percentage of under-treatment or an over estimation of disease estimated by questionnaire was present [31]. It is interesting to note that the incidence of asthma was not correlated with prevalence, and that a greater percentage of children needing a first time drug treatment was found in LHUs with a significantly lower percentage of chronically treated youths.

Moreover, neither the prevalence of asthma nor the prevalence of chronically treated youths correlated with the rate of hospitalisation.

## Conclusions

In conclusion, the analysis of drug prescriptions in children receiving asthma treatment highlighted a low compliance with the international guidelines, in particular in children treated with anti-asthmatics for the first time with LABA alone, raising questions on appropriateness of therapeutic choices. A further dynamic study will hopefully reveal modifications in therapeutic choices, as occurred in England [30].

## Abbreviations

ICS: inhaled corticosteroid; LABA: long acting beta adrenergic; LHU: Local Health Unit; LTRA: leukotriene receptor antagonist; NHS: National Health Service; PA: potential asthmatics; SABA: short acting beta adrenergic

## Competing interests

The authors declare that they have no competing interests.

## Authors' contributions

MB contributed to the conception and design of the study and drafted the manuscript. AC was involved in drafting the manuscript and revising it critically for important intellectual content. MS contributed to data analysis and performed the statistical analyses. AB, IF, and LM contributed to acquisition of data and general supervision. MB conceived the study, participated in its design, and gave final approval of the version to be published. All authors have read and approved the final manuscript

## Pre-publication history

The pre-publication history for this paper can be accessed here:

http://www.biomedcentral.com/1471-2466/11/48/prepub
